# Unexpected inflammation in the sympathetic ganglia in thromboangiitis obliterans: more likely sterile or infectious induced inflammation?

**DOI:** 10.1186/s12948-019-0114-2

**Published:** 2019-07-06

**Authors:** Behzad Mousazadeh, Hiva Sharebiani, Hossein Taheri, Narges Valizedeh, Bahare Fazeli

**Affiliations:** 1 Immunology Research Center, Inflammation and Inflammatory Diseases Division, School of Medicine, Mashhad University of Medical Sciences, Mashhad, Iran; 2 Surgery Department, Farabi Hospital, Mashhad, Iran; 3 Vascular Independent Research and Education, European Foundation, Milan, Italy

**Keywords:** Thromboangiitis obliterans, Buerger's disease, Sympathectomy, Sympathetic ganglia, Neurogenic inflammation, TLR4, TLR9, RAGE, HMGB1

## Abstract

**Introduction:**

The aim of this study was to determine if the inflammation of the sympathetic ganglia (SG) in thromboangiitis obliterans (TAO) is induced by an infectious pathogen inside or if it is a reactive sterile inflammation.

**Methods:**

For the purpose of this study, the gene expression of high-mobility group box 1 (HMGB1), toll-like receptor 4 (TLR4), toll-like receptor 9 (TLR9), and the receptor for advanced glycation end-products (RAGE) were evaluated on the complementary DNA (cDNA) of the SG tissues of 24 TAO patients and two controls with hyperhidrosis by real-time polymerase chain reaction (PCR) and analysed by the Pfaffl method.

**Results:**

The gene expression of HMGB1 and TLR9 increased by about 25- and 2-fold changes in the SG of the TAO patients, respectively. However, there was no change in the gene expression of TLR4 or RAGE.

**Conclusion:**

It appears that the inflammation in the SG of TAO patients is more likely a sterile inflammation, and its trigger may be mitochondrial DNA (mtDNA). Cadmium in cigarettes could be responsible for the induction of mtDNA release to the cell cytoplasm. In addition, the high expression of HMGB1 may play a role in the pathogenesis of TAO and may be responsible for both clinical manifestation of the disease and the imaging findings. Moreover, HMGB1 may be a target for treatment protocols for TAO. Further studies are highly recommended.

## Introduction

Thromboangiitis obliterans (TAO), or Buerger’s disease, is an inflammatory, thrombotic-occlusive, medium- and small-sized vasculitis that usually occurs in young males with a history of tobacco use [1]. The pathophysiology of the disease is not well understood. However, recently, the possible role of infectious pathogens as the trigger of TAO has been suggested [1]. For instance, the antibody for the main oral pathogen responsible for gingivitis, *Porphyromonas gingivalis* (*P. gingivalis*), has been detected in the sera of TAO patients, and this pathogen has also been isolated from vascular lesions using polymerase chain reaction (PCR) [2, 3]. However, since smoking might be responsible for gingivitis and related immunologic responses in TAO patients [4], this hypothesis has neither been confirmed nor disproved.

In addition to oral bacteria pathogens, since the 1980s, the possibility of Rickettsia infection has also been suggested as a trigger for TAO [5, 6]. In recent studies, Rickettsia infection was detected using PCR in 3 of 25 biopsies from a below-knee amputated limb in a TAO patient [7]. Following the study, the antibodies for the Rocky Mountain spotted fever (RMSF) group were detected in the sera of TAO patients using micro-immunofluorescence (MIF) [8].

According to our recent study, the infiltration of cytotoxic T lymphocytes and neutrophils in the sympathetic ganglia (SG) of TAO patients has been reported [9]. However, it is not yet known whether the inflammation in the SG is due to the presence of a pathogen and pathogen-associated molecular patterns (PAMPs) or if there is a sterile inflammation because of the presence of damage-associated molecular patterns (DAMPs).

Owing to the fact that, until recently, all of the infectious pathogens suggested as the trigger of TAO were gram-negative bacteria, the gene expression of toll-like receptor 4 (TLR4) as the receptor of innate immunity for the main PAMPs of gram-negative bacteria, so-called lipopolysaccharides (LPS), was evaluated in the sympathetic ganglia of TAO patients. With the released DNA due to cell injuries being the main inducer of sterile inflammation, toll-like receptor 9 (TLR9), as the main ligand for nucleic acids, was also evaluated. In addition, the gene expression of high-mobility group box 1 (HMGB1) and the receptor for advanced glycation end-products (RAGE) were evaluated.

HMGB1 is a chromatin structural protein of innate immunity, which usually increases during sterile inflammation and even infections with gram-negative bacteria [10]. During a sterile inflammation, HMGB1 can bind to extracellular DNA, which is released after traumatic shock or cell injury and, consequently, forms an HMGB1-DNA complex. This complex interaction with TLR9 can activate the myeloid differentiation primary response 88 (MyD-88) pathway and lead to the release of proinflammatory cytokines. RAGE binds directly to DNA and promotes its uptake into endosomes for activation of TLR9 as the main DNA-recognising receptor. RAGE can, thereby, sensitise cells to extra-cellular nucleic acids by decreasing the recognition threshold [11].

In addition, HMGB1 can be released upon LPS-induced TLR4 activation and, therefore, can bind LPS, even if only present in very small amounts, and carry it to the TLR4, leading to the release of proinflammatory cytokines.

In this study, the gene expression of HMGB1, TLR4, TLR9, and RAGE in the sympathetic ganglia of TAO patients was evaluated, and conclusions about the underlying mechanism of the inflammation were drawn according to the results (Table 1).

**Table 1:**
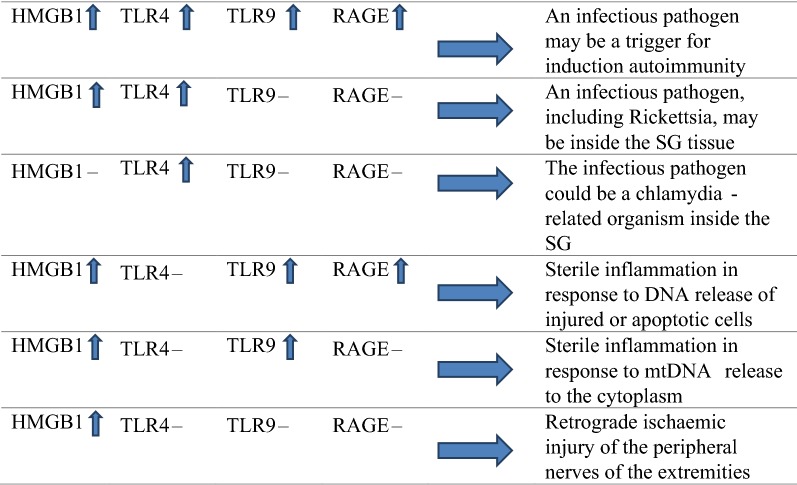
Different hypotheses according to different odds in the gene expressions of TLR4, TLR9, HMGB1, and RAGE

## Methods

The study was performed on the cDNA bank of SG tissue from 24 Caucasian male patients with a TAO diagnosis and SG tissue from two hyperhidrosis patients during 2009–2013. The mean age of the patients was 41.2 ± 1.3 years. All samples had a pathology report to confirm that the obtained tissue was from the sympathetic ganglia. The diagnosis of TAO was based on Olin’s criteria [12] or Shionoya’s criteria [13] with angiography confirmation. The demographic information, clinical presentation and high sensitivity C-reactive protein (hs-CRP) as an inflammatory laboratory marker of the patients has been summarized in Table 2.

**Table 2:** Demographic information, clinical presentation and hs-CRP) of 24 Caucasian male patients with TAO diagnosis

Patients	Age (year)	Age of cigarette smoking (year)	Smoking habit (Pack.Year)	Chief complaint	hsCRP (normal value < 3 µg/mL)
No. 1	38	28	10	Pain + non-healing ulcer	9.7
No. 2	47	16	62	Pain + toe gangrene	4
No. 3	39	19	40	Pain + toe gangrene	5
No. 4	43	35	8	Burning pain	16
No. 5	44	23	46	Pain + non-healing ulcer	49
No. 6	49	25	12	Pain + toe gangrene	9.2
No. 7	39	10	87	Pain + toe gangrene	18.6
No. 8	48	26	22	Pain + non-healing ulcer	7
No. 9	39	24	15	Pain + toe gangrene	2
No. 10	41	21	20	Pain + toe gangrene	10
No. 11	37	19	18	Burning pain	7.1
No. 12	32	17	15	Pain + toe gangrene	9
No. 13	35	23	12	Pain + toe gangrene	8.3
No. 14	30	14	16	Pain + toe gangrene	14
No. 15	48	7	123	Pain + toe gangrene	1.8
No. 16	49	14	140	Burning pain	41
No. 17	47	17	60	Pain + toe gangrene	0.5
No. 18	43	17	26	Pain + non-healing ulcer	8.3
No. 19	38	16	22	Pain + non-healing ulcer	12.5
No. 20	46	18	56	Pain + toe gangrene	21
No. 21	35	18	34	Pain + non-healing ulcer	1.5
No. 22	30	15	30	Pain + non-healing ulcer	1.8
No. 23	43	23	40	Burning pain	1.2
No. 24	50	30	10	Pain + non-healing ulcer	5.6

According to the side effects of sympathectomy, our biobank included no tissue from SG resection from healthy people. Thus, the cDNA of the SG of hyperhidrosis patients was used as the control for calculating the fold change of gene expression based on the Pfaffl method.

The gene expressions of TLR4 and HMGB1 were evaluated in the SG using the real-time PCR TaqMan^®^ method. The gene expressions of TLR9 and RAGE were evaluated in the SG using the real-time PCR SYBR^®^ Green method. The ribosomal protein large P0 (RPLP0) was considered the housekeeping gene for normalising the gene expression. The sequences of the primers for HMGB1, RAGE, TLR9, TLR4, and RPLP0 were borrowed from the primer bank of Harvard University, and the probes related to the paired primers were designed by Beacon Designer 7.0. The sequence similarity of the primers was checked with the basic local alignment search tool (BLAST), and the specificity of the primers for the intended genes was approved.

The Pfaffl method, also known as the delta–delta CT (∆∆CT) method, with efficiency correction [14] was used to calculate the gene expressions of HMGB1, RAGE, TLR9, and TLR4.

For TLR4, efficiency was 0.97; for TLR9, 0.92; for RAGE, 1.02; for HMGB1, efficiency was 0.80. For RPLP0, efficiency with TaqMan^®^ method was 0.96 and efficiency with SYBR^®^ Green method was 0.99.

For resection and banking of the tissue samples, the enrolled patients signed a broad consent form (ethical code for biobanking was MUMS-900133; ethical codes for evaluating HMGB1, RAGE, TLR9, and TLR4 gene expression publishing the data was 950047, 951598). None of the authors of this study had any conflict of interest.

## Results

**Fig. 1:**
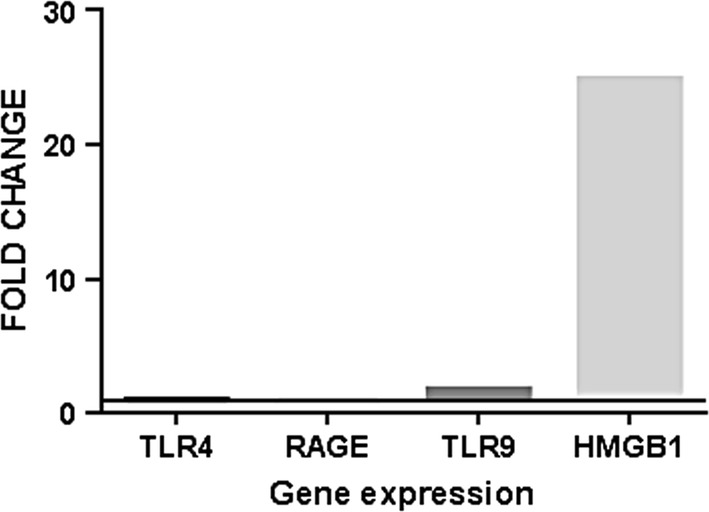
The gene expression of TLR4, RAGE, TLR9 and HMGB1 in SG tissue of TAO patients based on fold changes. The baseline of Y axis has been considered as onefold change which means no change

According to the Pfaffl method, no changes in the gene expression of TLR4 were found. However, the gene expression of HMGB1 was found to increase by approximately 24-fold changes. Therefore, the ratio of HMGB1 gene expression to TLR4 gene expression was also approximately 25-fold changes. Figure 1 shows the summarized results of the study.

$$\begin{aligned} {\text{TLR}}4 & = \frac{{{\left( {{\text{Efficiency TLR}}4} \right)^{{ - \Delta \Delta {\text{Ct}}_{{{\text{TLR}}4\left( {{\text{Mean control}} - {\text{Mean samples}}} \right)}} }} }}}{{{\left( {{\text{Efficiency RPLP}}0} \right)^{{ - \Delta \Delta {\text{Ct}}_{{{\text{RPLP}}0\left( {{\text{Mean control}} - {\text{Mean samples}}} \right)}} }} }}} \hfill \\ & = \frac{{{\left( {0.97} \right)^{{ - ( {27.57 - 27.50})}} }}}{{{\left( {0.96} \right)^{{ - ( {24.67 - 25.42})}} }} = 1.03{\text{-fold changes}}} \hfill \\ \end{aligned}$$

$$\begin{aligned} {\text{HMGB1}} & = \frac{{\left( {{\text{Efficiency HMGB1}}} \right)^{{ - \Delta \Delta {\text{Ct}}_{{{\text{HMGB1}}\left( {{\text{Mean}}\,{\text{control}} - {\text{Mean samples}}} \right)}} }} }}{{\left( {{\text{Efficiency RPLP}}0} \right)^{{ - \Delta \Delta {\text{Ct}}_{{{\text{RPLP}}0\left( {{\text{Mean}}\,{\text{control}} - {\text{Mean samples}}} \right)}} }} }} \hfill \\ & = \frac{{\left( {0.8} \right)^{{ - \left( {41.46 - 27.18} \right)}} }}{{\left( {0.96} \right)^{{ - \left( {24.67 - 25.42} \right)}} }} = 24.92{\text{-fold changes}} \hfill \\ \end{aligned}$$

$$\begin{aligned} {\text{TLR}}9 & = \frac{{\left( {{\text{Efficiency TLR}}9} \right)^{{ - \Delta \Delta {\text{Ct}}_{{{\text{HMGB1}}\left( {{\text{Mean}}\,{\text{control}} - {\text{Mean samples}}} \right)}} }} }}{{\left( {{\text{Efficiency RPLP}}0} \right)^{{ - \Delta \Delta {\text{Ct}}_{{{\text{RPLP}}0\left( {{\text{Mean}}\,{\text{control}} - {\text{Mean samples}}} \right)}} }} }} \hfill \\ & = \frac{{\left( {0.92} \right)^{{ - \left( {29.75 - 22.36} \right)}} }}{{\left( {0.99} \right)^{{ - \left( {20.14 - 18.58} \right)}} }} = 1.83{\text{-}}{\text{fold changes}} \hfill \\ \end{aligned}$$

$$\begin{aligned} {\text{RAGE}} & = \frac{{\left( {{\text{Efficiency RAGE}}} \right)^{{ - \Delta \Delta {\text{Ct}}_{{{\text{HMGB1}}\left( {{\text{Mean}}\,{\text{control}} - {\text{Mean samples}}} \right)}} }} }}{{\left( {{\text{Efficiency RPLP}}0} \right)^{{ - \Delta \Delta {\text{Ct}}_{{{\text{RPLP}}0\left( {{\text{Mean}}\,{\text{control}} - {\text{Mean samples}}} \right)}} }} }} \hfill \\ & = \frac{{\left( {1.02} \right)^{{ - \left( {32.72 - 32.38} \right)}} }}{{\left( {0.99} \right)^{{ - \left( {20.14 - 18.58} \right)}} }} = 0.98{\text{-fold changes}} \hfill \\ \end{aligned}$$

## Discussion

Although there is more than a century of TAO research from which to draw, the aetiology and pathophysiology of TAO is not yet understood. It is not yet even known whether the first event in TAO is thrombosis or inflammation (angiitis) [1]. However, several studies have shown that infectious factors are a likely trigger for TAO development [2–8]. Notably, the clues that point to the role of infectious pathogens in TAO suggest gram-negative pathogens.

Recently, we discovered an infiltration of neutrophils and cytotoxic T lymphocytes in the SG of TAO patients [9]. In the current study, we wanted to discover if the infiltration of inflammatory cells in the SG of the patients was due to the presence of a pathogen inside the SG tissue or whether there was a reactive inflammation due to irritation of the peripheral nerves in ischaemic tissue or neural cell injuries in SG.

For this purpose, we evaluated the gene expression of HMGB1 as an inflammatory marker that can increase both due to release of DNA from injured cells (sterile inflammation) and also in response to gram-negative bacteria [10, 11]. Also, the gene expression of TLR4 was evaluated because it is a receptor of innate immunity for LPS and can increase due to infection with gram-negative bacteria [15]. Therefore, we hypothesised that, if the gene expression of both TLR4 and HMGB1 increased without change in the gene expression of TLR9 or RAGE, then an infectious pathogen, including Rickettsia, could be inside the SG tissue and be responsible for the inflammation in the sympathetic ganglia. If only the gene expression of TLR4 increased without any change in the gene expression of HMGB1, the infectious pathogen might be a chlamydia-related organism, in which there is a serological cross-reactivity between Rickettsia and chlamydia [16]. If HMGB1 increased without any increase in TLR4 gene expression but with an increase of TLR9 or RAGE expression, this would demonstrate an endogenous source of inflammation in the SG, probably following cell injuries that lead to DNA release [17]. However, if HMGB1 increased without any change in the gene expression of TLR4, TLR9, or RAGE, then it might be induced by the retrograde ischaemic injury of the peripheral nerves of the extremities [18]. Finally, if the gene expression of all studied genes—including TLR4, TLR9, RAGE, and HMGB1—increased, this could indicate the role of neutrophil extracellular traps (NETs) as a response to a gram-negative bacteria trigger, which could be the source of extracellular DNA for induction of sterile inflammation (infectious induced autoimmunity) [19].

Our results demonstrated that the gene expression of HMGB1 and TLR9 increased about 25- and 2-fold changes in the SG of TAO patients, respectively. However, there was no change in the gene expression of TLR4 or RAGE. Therefore, the most appropriate conclusion was that the inflammation inside the SG could be a sterile inflammation due to the presence of nucleic acids, such as DNA, but not due to ischaemic injuries of the nerve end terminals.

**Fig. 2:**
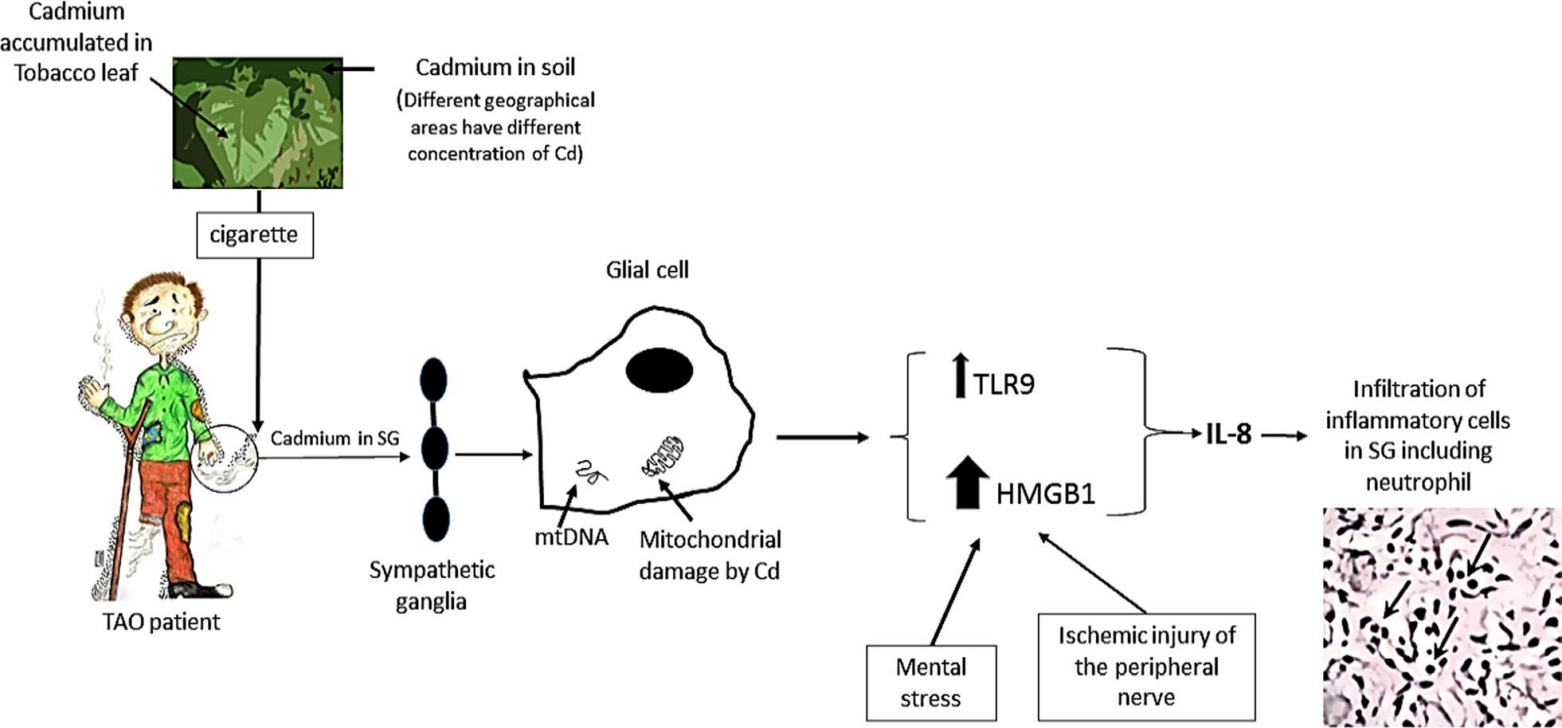
The figure shows how the possible injuries of the cells in the SG of TAO patients may lead to the release of DNA, increase the gene expression of HMGB1, and also activate the TLR9 pathway

Next, we will explain how the possible injuries of the cells in the SG of TAO patients may lead to the release of DNA, increase the gene expression of HMGB1, and also activate the TLR9 pathway (Fig. 2).

One of the most important and dangerous components of cigarettes is cadmium [20]. Cadmium in the soil can be accumulated in tobacco leaves [21, 22]. Notably, cadmium can accumulate in the nervous system, including in the SG [23, 24]. Cadmium tends to influence mitochondrial DNA more than nuclear DNA by mtDNA cleavage, inhibit adenosine triphosphate (ATP) synthesis, and, consequently, induce apoptosis [25, 26]. Moreover, mtDNA damage, the reactive oxidative stress that is induced by cigarette smoking, enhances the release of mtDNA into the cytoplasm and, consequently, induces the expression of HMGB1 to make an HMGB1-mtDNA complex that activates cytosolic TLR9 [27]. Following TLR9 activation, interleukin 8 (IL-8), a neutrophil chemoattractant, is released, which may explain the neutrophil infiltration in the SG of the patients [9, 25].

However, if HMGB1 makes a complex with extracellular DNA due to cellular damage, this complex will require RAGE for internalisation and activation of TLR9 [28]. Owing to the fact that the gene expression of RAGE was almost unchanged in the current study, mtDNA may play an important role in initiating inflammation by activating TLR9, since mtDNA is present in cytosol and does not need to internalisation by RAGE.

In addition, according to animal-based studies, HMGB1 can be upregulated due to retrograde ischaemic injury of the peripheral nerves of the extremities or due to response to neuropeptide Y, which is usually overexpressed in the sympathetic system following psychological stress [18, 29]. Therefore, the 25-fold increase in the gene expression of HMGB1 may have several triggers, including release of mtDNA, mental stress, or ischaemic injury of the nerves due to vascular occlusion. The hypothesis.

However, aside from findings concerning the underlying trigger of inflammation in the SG of TAO patients, one of the precise findings of this study was the high gene expression of HMGB1 in the SG of TAO patients. HMGB1 is a pro-angiogenic factor, which can induce the production of vascular endothelial growth factor (VEGF) [16]. Since it has been found that the corkscrew collaterals in TAO arteriography are vaso-nervorum, not vaso-vasorum [30], the high expression of HMGB1 may be responsible for the formation of corkscrews in TAO.

Moreover, owing to the fact that HMGB1 induces both chemotaxis of neutrophils and inflammatory cells and platelet activation [31, 32], it is not yet known whether HMGB1 could be transported alongside the sympathetic nerves and released from their end terminals. Because a high serum level of HMGB1 in TAO patients has also been reported [33], it is possible that HMGB1 does not release from the end terminals of the sympathetic nerves. Instead, the possible release of neuropeptide Y due to psychological stress may induce expression of HMGB1 in the macrophages of the tissues receiving the sympathetic nerve supply [34]. Thus, it could be responsible for inflammation, thrombus formation at the site of vascular denervation, and corkscrew formation.

On the other hand, TAO might be a type of neurogenic inflammation. This hypothesis could explain the skip lesions and corkscrews in the arteriography of TAO patients. It could also explain the close relationship of the patients’ clinical signs and symptoms with smoking. In addition, it could explain TAO flare-up after mental stress and signs and symptoms related to vasoconstriction, including cold sensation of the toes, cold sensitivity, and Raynaud’s phenomenon, as the early signs of TAO. Moreover, it seems there is a close interaction between nervous stress and inflammation with consequent vascular damages [35]. However, more investigation is needed to prove or disprove this hypothesis.

## Conclusion

The first conclusion, based on the 25-fold change higher gene expression of HMGB1 in the SG of TAO patients and also the high gene expression of TLR9, without any change in the gene expression of TLR4 or RAGE, could indicate a sterile and reactive inflammation in the SG in response to the release of mtDNA to the cytoplasm. In addition, the high expression of HMGB1 may play a role in the pathogenesis of TAO and may be responsible for clinical manifestation of the disease, the imaging findings, and the close relationship of the disease with smoking. Moreover, HMGB1 may be a target for treatment protocols for TAO, that eliminates HMGB1-mediated inflammation without interfering with adaptive immune responses [36]. Further studies are highly recommended.

## Data Availability

The cDNA samples of the sympathetic ganglia are available and banked in the biobank of Inflammation and Inflammatory Diseases Research Center located in Immunology Department, Medical school, Pardis Campus, Mashhad University of Medical Sciences, Azadi Sqr., Mashhad, Iran. The data and all the curves and cyclic threshold (CT) of the results are available and will be sent upon the request from the journal.
